# Tracking the Luminal Exposure and Lymphatic Drainage Pathways of Intravaginal and Intrarectal Inocula Used in Nonhuman Primate Models of HIV Transmission

**DOI:** 10.1371/journal.pone.0092830

**Published:** 2014-03-25

**Authors:** Jeremy Smedley, Baris Turkbey, Marcelino L. Bernardo, Gregory Q. Del Prete, Jacob D. Estes, Gary L. Griffiths, Hisataka Kobayashi, Peter L. Choyke, Jeffrey D. Lifson, Brandon F. Keele

**Affiliations:** 1 Laboratory Animal Sciences Program, Leidos Biomedical Research, Inc., Frederick National Laboratory, Frederick, Maryland, United States of America; 2 Molecular Imaging Program, National Cancer Institute, Bethesda, Maryland, United States of America; 3 The AIDS and Cancer Virus Program, Leidos Biomedical Research, Inc., Frederick National Laboratory, Frederick, Maryland, United States of America; 4 Clinical Monitoring Research Program, Leidos Biomedical Research, Inc., Frederick National Laboratory, Frederick, Maryland, United States of America; Harvard Medical School, United States of America

## Abstract

Over 80% of sexual HIV-1 transmissions originate from a single viral variant, but the underlying basis for this transmission bottleneck remains to be elucidated. Nonhuman primate models of mucosal virus transmission allow opportunities to gain insight into the basis of this mucosal bottleneck. We used simulated inocula consisting of either non-infectious vital dye or contrast dye with non-invasive magnetic resonance imaging (MRI) to visualize mucosal exposure and passive lymphatic drainage patterns following vaginal and rectal exposures in Indian origin rhesus macaques. Results revealed a limited overall distance of dye coverage from the anal verge following 1 ml (n  = 8) intrarectally administered, which greatly increased with a 3 ml (n = 8) volume. Intravaginal dye exposure using 2 ml revealed complete coverage of the mucosa of the vagina and ectocervix, however dye was not detectable in the endocervix, uterus, fallopian tubes or ovaries in nuliparous sexually mature rhesus macaques (n = 9). In addition, following submucosal and intranodal injections of vital dye or MRI contrast dye in the rectum (n = 9), or distal and proximal vagina (n = 4), the lymphatic drainage pathways were identified as first the internal then common iliac chain followed by para-aortic lymph nodes. Drainage from the distal descending colon (n = 8) was via the para-colonic lymph nodes followed by the inferior mesenteric and para-aortic lymph nodes. Analysis after vaginal challenge with infectious SIVmac239 followed by euthanasia at day 3 revealed a pattern of viral dissemination consistent with the imaging results. These results provide insights into potential patterns of viral dissemination that can help guide efforts to better elucidate the earliest events of virus transmission and potential intervention strategies.

## Introduction

Worldwide, there are an estimated 35.3 million people who are currently living with human immunodeficiency virus (HIV) infection [1]. The CDC estimates that approximately 49,000 new cases occurred in 2011 in the US alone, with the vast majority of transmissions (∼44,000) occurring across a mucosal barrier [2]. While homosexual transmission (male to male sexual contact) is the most common route of transmission in the US, heterosexual transmission is the predominant route of viral transmission globally [3]. The rate of HIV acquisition is affected by multiple factors that include transmission route, frequency of sexual contact, and the viral load of the infected partner. Recently, genetic analyses of primary infections have shown that in nearly 80% of sexual transmissions, infection in a new host is established by a single viral variant from among the diverse quasispecies present in a typical transmitting partner [4]–[9]. However, viral transmission rates increase dramatically when ulcerative or inflammatory conditions affecting the exposed mucosa are present [5]. Taken together, these data suggest that an intact mucosal barrier plays a key role in inhibiting HIV transmission during sexual exposure with the vast majority of the HIV inoculum blocked at the mucosal surface or otherwise restricted from establishing systemic, productive infection [10]. Understanding the exact anatomic location of the viral inoculum and events immediately following exposure to infectious virus could provide additional insights into transmission risks and potential intervention strategies.

Access to relevant mucosal tissues at critical time points following exposure is extremely limited in HIV infected subjects. Experimental SIV infections of rhesus macaques (Macaca mulatta) have been used for decades to study various aspects of primate lentiviral infections, including transmission, pathology, prevention, and treatment. Recently, a great deal of attention has been paid to the isolate or type of virus used in this model, the amount of virus in the inoculum, the number and frequency of inoculations, and the potential for non-viral constituents to influence infectivity [11]–[16]. Less attention has been paid to physical and anatomical considerations, including the volume of the inoculum, position of the animal during exposure, duration of exposure, the amount of mucosa exposed to the inoculum, the sites at which viral transmission may occur, and the relevant draining lymphatic tissues involved in systemic dissemination of the infection. The potential influence of these factors on transmission is typically not even considered in most studies of transmission or prophylactic interventions. Though methodology is not formally standardized across laboratories, a typical mucosal inoculation involves the atraumatic application of 1 ml of appropriately diluted viral inoculum for intrarectal challenges and up to a 2 ml volume for intravaginal challenges. Evaluation of the outcome of such challenges has been based on infection endpoints without regard for whether such inoculation approaches provide consistent exposure of the mucosa to the viral inoculum. Inconsistent mucosal exposure to the inoculum has the potential to result in increased inter-animal variability, particularly in single or repeated challenge studies using inocula titered to yield transmission of a single or only a few viral variants, as is commonly done in current studies, to authentically recapitulate this clinically relevant feature of HIV transmission.

Here, we employed a simulated challenge strategy using either a magnetic resonance imaging (MRI) contrast dye followed by MRI imaging or methylene blue dye to simulate typical mucosal inoculation procedures, followed by visual inspection at necropsy to address the fundamental questions of: 1) how far can the inoculum spread and to what extent does it contact mucosal surfaces? and 2) what are the first lymphatic pathways involved in dissemination of virus or virally infected cells from these initial sites of viral exposure and infection? We show here that a 3 ml intrarectal challenge provided significantly greater and more consistent mucosal coverage than a typical 1 ml exposure. For vaginal exposure, a 2 ml volume in sexually mature, nulliparous rhesus macaques resulted in the inoculum contacting the entire vaginal vault, but we found no evidence of penetration into the cervix regardless of phase (follicular, luteal, or active menses) of the menstrual cycle. Furthermore, these dye studies indicated that lymphatic drainage from vagina and rectum is via the internal and then common iliac nodes before draining into the para-aortic chain. In contrast, the distal descending colon drains via the para-colonic and inferior mesenteric lymph nodes, which eventually drain into the para-aortic chain. Finally, we implemented the same inoculation protocol employed in the simulated challenges for a vaginal challenge using replication competent SIVmac239 and showed a pattern of viral dissemination consistent with the imaging results, confirming the validity of our approach. Future studies mapping early viral or host events following mucosal challenge or preclinical vaccine studies should consider sampling sites and tissues identified here as likely routes of viral transmission and systemic dissemination.

## Materials and Methods

### Animals

All animals were housed at the National Institutes of Health (NIH) in accordance with the Association for the Assessment and Accreditation of Laboratory Animal Care (AAALAC) standards and all procedures were performed according to protocols approved by the Institutional Animal Care and Use Committee of the National Cancer Institute (Assurance #A4149-01). Animals were maintained in Animal Biosafety Level 2 housing according to the provisions of the 5th edition of the Biosafety in Microbiological and Biomedical Laboratories with a 12:12-hour light:dark cycle, relative humidity 30% to 70%, temperature of 23 to 26°C and all animals were observed twice daily by the veterinary staff. Filtered drinking water was available ad libitum, and a standard commercially formulated nonhuman primate diet (Purina Labdiet 5045 “High Protein Monkey diet”, PMI Nutrition International, St. Louis, MO) was provided thrice daily and supplemented 3–5 times weekly with fresh fruit and/or forage material as part of the environmental enrichment program. Environmental enrichment: Each cage (Allentown, Inc., Allentown, NJ) contained a perch, two portable enrichment toys, one hanging toy, and a rotation of additional items (including stainless steel rattles, mirrors, and challenger balls). Additionally the animals were able to listen to radios during the light phase of their day and were provided with the opportunity to watch full-length movies at least three times weekly. All animals used on this study were nuliparous sexually mature Indian origin rhesus (M. mulatta) between 3.5 and 13.5 kg. In total 24 macaques were used across the various groups. For MRI a total of twelve imaging sessions were performed utilizing 6 different macaques examined between 3 and 8 pm. All animals were anesthetized with ketamine (10 mg/kg) and dexmedetomidine (25 ug/kg) followed by intubation and administration of isoflurane (1–3%). They were monitored throughout the process by a veterinarian and recovered without incident. All 24 animals were utilized in conjunction with other studies and any euthanasia performed (pentobarbital 80 mg/kg after sedation as above) was as a result of reaching study endpoints on the primary protocol and occurred between 6 and 9 am. Prior to use, all animals were tested and confirmed seronegative for macacine herpes virus 1 (herpes B), Simian Immunodeficiency Virus (SIV), Simian T Lymphotropic Virus (STLV), and Simian Retrovirus (SRV) and were negative for SRV by PCR as well. Additionally all animals were treated with enrofloxacin (10 mg/kg once daily for 10 days), paromomycin (25 mg/kg twice daily for 10 days), and fenbendazole (50 mg/kg once daily for 5 days) followed by weekly fecal culture and parasite exams for 3 weeks to ensure they were free of common enteric pathogens. At least a 4 week post-treatment period allowed time for stabilization of the microbiome prior to use in this study. Menstrual cycles were monitored by daily swabbing of the vaginal vault with a cotton-tipped applicator looking for evidence of menstrual bleeding after acclimating the animals to accept the procedure using positive reinforcement techniques.

This study utilized animals available after completion of other studies, with group sizes based in part on the availability of animals. Animals were randomized across the different treatment groups. Group sizes were as follows, i) 1 ml methylene blue dye administered intraluminally into the rectum n = 5; ii) 1 ml MRI contrast administered intraluminally into the rectum n = 3; iii) 3 ml methylene blue dye administered intraluminally into the rectum n = 6, iv) 3 ml MRI contrast administered intraluminally into the rectum n = 2; v) Methylene blue dye administered intraluminal into the vagina n = 6; vi) MRI contrast administered intraluminal into the vagina n = 3; vii) methylene blue dye administered submucosally into the rectum n = 5; viii) MRI contrast administered submucosally into the rectum n = 4; ix) methylene blue dye administered submucosally into the descending colon n = 5; x) MRI contrast administered submucosally into the descending colon n = 3; xi) methylene blue dye administered submucosal into the vagina n = 2; xii) MRI contrast administered submucosal into the vagina n = 2; and xiii) SIV infection n = 1. This represents 47 independent analyses performed on these 24 rhesus macaques. Where animals were utilized in more than one group an appropriate washout period was employed as noted below.

### Administration of Intraluminal Inoculum

Simulated challenges were performed using 1 ml or 3 ml volumes for intrarectal administrations and 2 ml for intravaginal administrations of either methylene blue dye (1%w/v in RPMI-1640) or Gadofosveset trisodium (Ablavar Lantheus Medical Imaging, Billerica, MA, USA), which was purchased commercially (diluted with saline to a Gadolinium concentration of 40 mM). Animals were placed at an approximately 25° to 45° down angle in an inverted Trendelenburg position (i.e. the animal’s pelvis was elevated above its head with its sternum against the table) and the simulated challenge was performed using 1cc slip tip syringes (BD Biosciences) with a small amount of non-bacteriostatic, single-use, sterile lubricant applied to the exterior, taking care to not occlude the tip. In 4 instances, animals were subjected to only approximately a 25° down angle (DC66, ZE72, ZB35, ZG04). As there were no significant differences based on the degree of down angle within these groups the results of all animals were combined for analysis.

Methylene blue administration was followed 20 minutes later by euthanasia and the extent of mucosal coverage was assessed at necropsy after en bloc removal of the large intestine or the entire female reproductive tract for the IR and Ivag groups, respectively. No attempt was made to alter the natural contracted or relaxed state of the excised tissue. Results were documented by digital photography, including before and after removal of feces by rinsing in PBS for the IR inoculated animals. Ablavar® administration was followed 20 minutes later by imaging as detailed below.

### Contrast Agent Injections

Lymphatic drainage was further mapped by injecting 0.05 ml Gadolinium (Gd) G5-DOTA dendrimer (diluted with saline to a Gd concentration of 10 mM) through a 27–30 G needle into the submucosa of the vaginal wall (∼1.5–3 cm from the vaginal introitus), rectum (∼1.5–3 cm from the anus) or distal descending colon (∼10–13 cm from anus). For each challenge route two injections of contrast were placed at sites aproximately180° apart circumferentially, for a total injected volume of 100 μl. Injections formed a visible bleb on the mucosal surface indicating successful injections into the submucosa. In 5 animals studied post-mortem, methylene blue dye was injected submucosally or subserosally followed by intranodal injections into lymph nodes that showed dye uptake after the submucosal or subserosal injections, to identify downstream lymphatic pathways. Some animals undergoing MRI were utilized in multiple treatment groups after a sufficient washout period of the MRI contrast agents between groups to ensure that no contrast from previous sessions was visible on baseline images taken prior to the administration of the contrast agent for the subsequent treatment. Injections occurred outside of the magnetic field of the MRI and animals were immediately returned for imaging following dye injection requiring no more than 5 minutes until imaging started. Imaging sessions took 10 minutes each.

### Virus Inoculation

To assess the relevance of the MR and methylene blue imaging results to actual infectious virus challenges, a single 2 ml intravaginal challenge was performed in a rhesus macaque with 3×10^5^ TCID_50_ of transfection-derived SIVmac239, utilizing methodology identical to that employed in the simulated challenges. This was followed by necropsy and extensive tissue collection 3 days later, with molecular analysis of specimens for the presence of SIV nucleic acids.

### In-vivo MR Imaging

Magnetic resonance imaging (MRI) studies were conducted at 3 Tesla (T) (Achieva, Philips, The Best, Netherlands). A 3D T1 weighted (T1W) sequence with fat suppression was acquired with a 32 channel cardiac coil (Philips Medical Systems, Best, Netherlands) before and after administration of the contrast agents (intraluminal with gadofosveset trisodium or intramucosal with Gd G5-DOTA dendrimer injections). All MR images were obtained using the Constant LEvel AppeaRance (CLEAR) option after acquiring a coil reference scan. Two different MRI protocols based on the size of the monkeys were used and basic parameters were for monkeys less than 5 kg, TR and TE of 7.5 and 3.3msec, matrix of 600×198, field of view 240×99×72 mm, flip angle 25°, Voxel size 0.25 mm^3^, and acquisition time of 466sec. For animals larger than 5 kg, the basic parameter were TR and TE of 6.9 and 3.3msec, matrix of 788×199, field of view 300×140×90 mm, flip angle 25°, Voxel size 0.35 mm^3^, and acquisition time of 381sec.

### Image Analysis

All MR image analyses were performed by multiple readers on a commercially available workstation (Extended MR WorkSpace [EWS], Philips, The Best, Netherlands) or with OsiriX DICOM viewer v5.6. For each imaging session, baseline and post-injection MR images were evaluated together to evaluate the contrast enhancement in eight lymph node groups (inguinal, external iliac, internal iliac, common iliac, para-aortic, inferior mesenteric, left-colic, and para-colonic).

### In Situ Hybridization

We utilized a novel next generation RNA in situ hybridization technology, RNAscope, for SIV in situ hybridization. A series of target probes were designed to hybridize to SIV viral RNA utilizing the SIV template genome D01065.1 SIVMM32H in gag, vif, pol, tat, env, vpx, vpr, nef, rev genes. The target probe design strategy was described previously [17]. Briefly, each target probe contains an ∼25-base region complementary to the SIV plus-RNA strand (transcribed transcripts or whole transcribed genome) of each gene, a spacer sequence, and a 14-base tail sequence (conceptualized as a Z). A pair of target probes (double Z), each possessing a different type of tail sequence, hybridize contiguously to a target region (∼50 bases). The two tail sequences together form a 28-base hybridization site that binds to a signal preamplifier, which initiates a signal amplification cascade via sequential hybridization, similar to the branched DNA (bDNA) method described previously [18], followed by chromogenic enzymatic detection (alkaline phosphatase using Fast Red substrate). This approach targets ∼4 kb of the SIV genome. The double-Z probe design strategy ensures superior background control because of the extraordinarily low probability that nonspecific hybridization events will juxtapose a pair of target probes along an off-target mRNA molecule to form the 28-base hybridization site for the preamplifier, and also because a single 14-base tail sequence will not bind the preamplifier with sufficient strength to result in successful signal amplification. In situ hybridization was performed on at least 15 subjacent sections of lymph nodes (iliac, inguinal, axillary and mesenteric) 15–20 μm apart from each other, prepared from a RM sacrificed 3 days after atraumatic intravaginal SIVmac239 challenge and two SIV- RMs. In addition to the female genital tract (vagina and cervix), rare SIV vRNA+ cells were only found within one iliac lymph node in the RM sacrificed 3 days post vaginal challenge. These rare SIV vRNA+ cells were found within at least 3 iliac lymph node sections. To confirm specificity of ISH, non-specific (sense) probes were used as well as specific probes in SIV-negative animals.

### Statistical Analysis

A two-tailed student T-test was used to compare length of mucosa covered by dye and overall dye surface area coverage following intrarectal administration 1 ml or 3 ml of dye.

## Results

### Modeling Intrarectal Inoculation with Methylene Blue Dye and MRI Contrast

**Figure 1:**
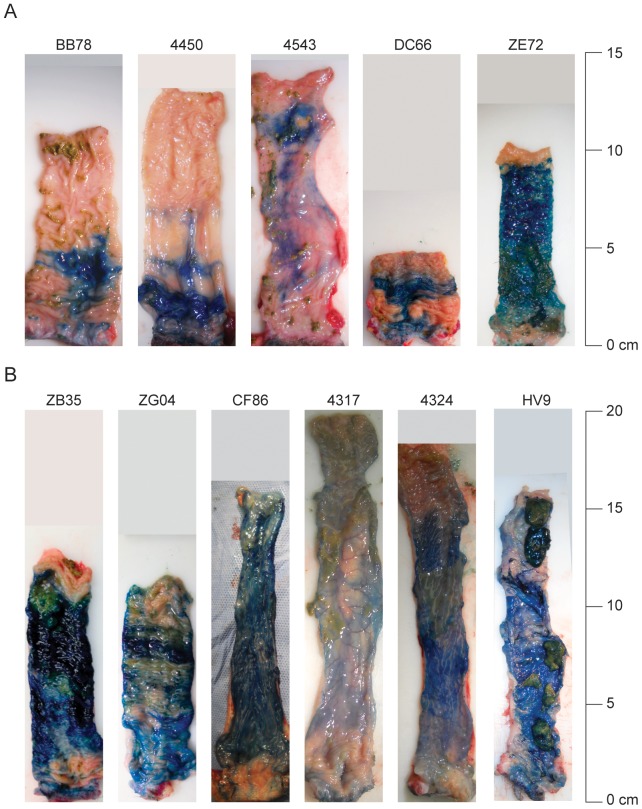
Methylene blue dye staining of rectum and descending colon following a 1 ml (A) or 3 ml (B) simulated challenge. Twenty minutes following dye exposure, animals were necropsied and rectum/distal colon was removed en block. Once fecal matter was gently cleared from tissue, stained mucosal tissue was photographed to assess both completeness coverage and the maximal distance of dye coverage from the anal verge.

To evaluate luminal mucosal coverage following an intrarectal mucosal inoculation approach typically used in NHP models of AIDS, we exposed 5 rhesus macaques to 1 ml of methylene blue dye 20 minutes prior to scheduled euthanasia. Animals were placed at an approximately 25-45° down angle in an inverted Trendelenburg position and dye was atraumatically administered intrarectally. At necropsy, the rectum and distal descending colon were examined in situ for extent of dye distribution. Following en block dissection, fecal matter was gently cleared from sample and stained mucosal tissue was photographed to assess both completeness of dye coverage and maximum distance of dye staining, measured from the anal verge with no attempt to alter the natural extended or contracted state of the tissue (Fig. 1A). In 4 of 5 animals, dye staining of the mucosal surface was spotty, lacking uniform coverage. The measured distance of detectable dye staining was 7.7 cm (SD±3.5) from the anal verge with a range of 3.5–13 cm. In 3 of 5 animals, the maximum length of detectable dye was less than 7 cm and was confined anatomically to the rectum, while the remaining 2 animals (4543 and ZE72) had dye reaching the distal descending colon. The consistency and amount of feces varied between animals and likely contributed to non-uniform mucosal exposure to the introduced dye.

Based on the highly variable nature of mucosal exposure following the 1 ml inoculations, we tested whether a 3 ml inoculum volume might mitigate the impact of intraluminal feces and result in greater and more consistent mucosal exposure to the dye-containing simulated inoculum. An additional 6 animals were exposed to 3 ml of methylene blue followed by necropsy with tissue harvest 20 minutes later. Again, following en block dissection, fecal matter was gently cleared from tissue and stained mucosal tissue was photographed to assess both completeness of dye coverage and the maximum distance of detectable dye staining from the anal verge (Fig. 1B). We noted in all 6 animals an increase in the consistency of dye coverage and a greater combined maximum distance of dye staining from the anal verge compared to animals receiving the 1 ml dye inoculum. The measured length of detectable dye staining was 14.9 cm (SD±2.8) and a range of 11–18 cm, which was significantly greater than 7.7 cm for the 1 ml inoculum (p<0.004). Using imaging software, the total stained area was determined for each animal with no attempt to compensate for lumenal folds. The 3 ml inoculum had significantly more stained area with a mean of 27.5 cm^2^, compared to the 1 ml exposed animals with a mean of 13.3 cm^2^ (p<0.003). We noted that firm feces, found in animals 4450, DC66, ZG04, and HV9, tended to prevent coating of the mucosa in specific areas, while softer feces found in animals 4543, 4317, and 4324, tended to mix with the dye resulting in more even but lighter coating indicative of a dilution of the dye. Although there were both firm and soft feces in each group, the overall mucosal coverage was still significantly higher in the 3 ml inoculated animals than the 1 ml inoculated animals indicating that higher volume viral inoculations may provide greater and more consistent mucosal exposure than the traditional 1 ml challenge and could be used in future SIV infection studies.

**Figure 2:**
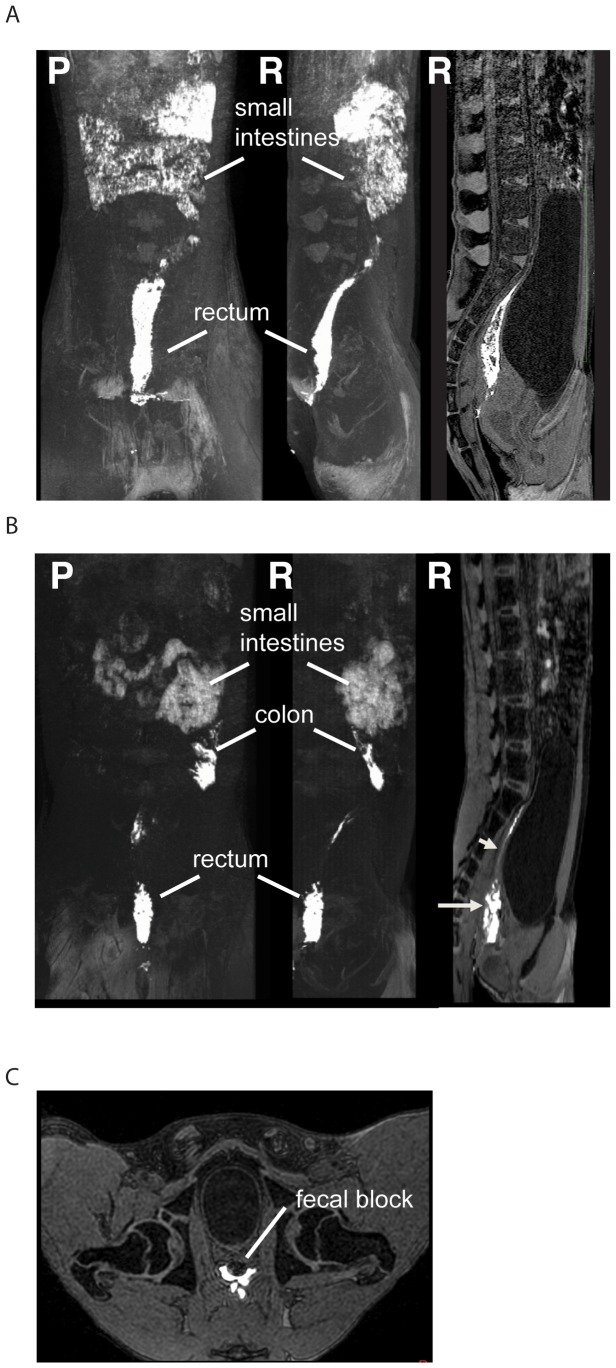
Magnetic resonance imaging to visualize contrast dye introduced intrarectally in two rhesus macaques exposed to a 1(A) or 3 ml (B) inoculum. In both A and B, the left two images are maximum intensity projections representing a 3D reconstruction, while the right panel shows a cross-sectional image. The orientation of the image is indicated for each panel at top left with P indicating posterior towards the reader and R the right side towards reader. Gadofoveset trisodium contrast dye was administered intrarectally and 20 minutes later animals were returned to a recumbent position and MRI was performed. (A) Although highly variable between animals, the 1 ml exposure shown here shows robust inoculum penetration including within the distal descending colon. Panel B represents a 3 ml exposure with robust and thorough dye coverage in the rectum and in the descending colon. Bladder compression of the distal descending colon results in a thin layer of dye between these two sites, which, though difficult to see, likely coats the compressed mucosa as noted by the short arrow in the far right panel. Also present in this panel is apparent mixing of feces with the contrast agent represented by the long arrow. Panel C represents cross-sectional image showing solid fecal matter blocking contrast from contacting the mucosa. Although background contrast was detected in the small intestines due to animal diet, contrast dye in the rectum and descending colon is clearly visible. These images were selected to represent the variable outcomes of the 5 animals tested by MRI for luminal exposure.

As a correlative study in living, sedated rhesus macaques, we modified the standard use of magnetic resonance imaging (MRI) to allow for visualization over time of contrast dye introduced intrarectally. Animals were again placed in an inverted Trendelenburg position and 1 ml or 3 ml of gadofoveset trisodium solution was atraumatically administered. Animals were kept in a downward position for 20 minutes and then rotated into a recumbent position for the MRI procedure. Contrast dye was clearly detectable in the rectum and descending colon in 1 ml (Fig. 2A and Movie S1) and 3 ml (Fig. 2B and Movie S2) inoculated animals. Interestingly, one can clearly discern the impact of hard stool within the lumen of the GI tract which blocks direct dye contact with the mucosa (Fig. 2C). The extent of contrast dye coverage was determined by measuring the X, Y and Z coordinates within the rectum and distal descending colon. For the 3 animals receiving 1 ml of contrast dye, the measured length of detectable dye staining was 9.3 cm (SD±4.0) with a range of 5–13 cm. This is less than the 14.2 cm (SD±2.6) maximum (range of 12.3–16 cm) for the two animals that received 3 ml of contrast dye. These measured values were consistent with our earlier methylene blue dye measurements and did not change over the course of the three repeated MRI scans at approximately 10-minute intervals. When both approaches were combined, we found that the average maximum distance from the anus for dye exposure following 3 ml inoculation was 14.7 cm (SD±2.6), which is significantly greater than 8.3 cm (SD±3.6) seen with 1 ml dye inocula (p = 0.001). Furthermore, the overall inoculum coverage and circumferential spread appears to be greater with 3 ml challenges compared to traditionally used 1 ml inoculations, suggesting that viral inoculations with 3 ml may increase reproducibility in viral challenges especially when the viral dose is limited and the risk of losing a significant fraction of the inoculum in feces is high.

### Modeling Intravaginal Inoculation with Methylene Blue Dye and MRI Contrast

**Figure 3:**
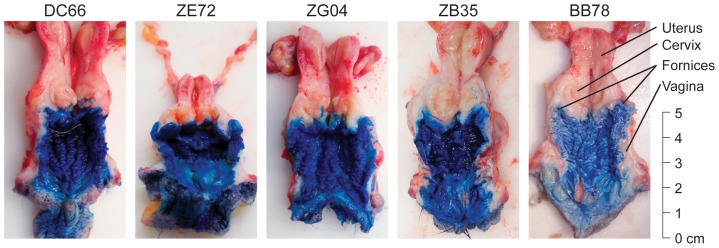
Methylene blue dye staining of female genital tract following a 2 ml inoculum challenge. Twenty minutes following dye exposure, animals were necropsied and the entire female genital tract was extracted en block and dissected. The tissue was photographed to assess both completeness of dye coverage and length of distance from the vaginal introitus. Overall there was complete dye staining in the vaginal vault, but stain was not detected in the cervix, uterus, or ovaries.

The female genital tract (FGT) represents a very important and highly relevant site of AIDS virus transmission. Despite a number of studies seeking to determine the precise anatomical location of transmission within the FGT, the respective roles of transmission across the vaginal and cervical epithelia remain unclear. To better understand the anatomic and physical considerations in this process, we utilized both methylene blue dye and MRI contrast dye to determine the extent of inoculum coverage of the vagina and penetration into the cervix and uterus. Sexually mature rhesus macaques were placed at an approximately 25° down angle in an inverted Trendelenburg position and 2 ml of dye was atraumatically administered intravaginally to 5 non-menstruating (Fig. 3) and 1 actively menstruating (Fig. S1) animals. For methylene blue dye studies, animals remained in this position for 20 minutes prior to euthanasia. Following necropsy and dissection, stained mucosal tissue was photographed for completeness of dye coverage and distance from vaginal introitus was measured. In all 6 animals, we discovered complete coverage of the mucosa of the vagina including the vaginal fornix and the ectocervix. However, there was no evidence of dye penetration into the endocervical canal, uterus, fallopian tubes, or ovaries. This lack of penetration was noted in all animals regardless of menstrual phase.

**Figure 4:**
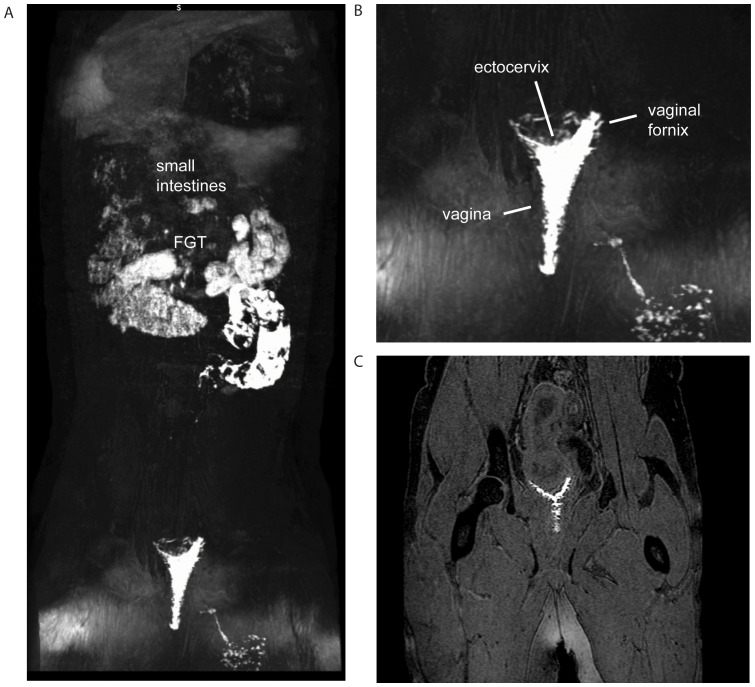
Magnetic resonance imaging to visualize contrast dye introduced intravaginally. 2-sectional image. The orientation of all images is posterior towards the reader. Panel A shows a wider view of the animal including the entire abdomen while panel B highlights the pelvis at greater magnification. Panel C is a 2D image demonstrating the inability to detect contrast penetration into the cervix/uterus despite the high degree of anatomic resolution that can be achieved by MRI. Although significant background contrast was detected in the small intestines due to minerals present in the animal diet, contrast dye in the vagina is clearly visible. These images were selected to represent the highly consistent vaginal exposure in 3 animals examined by MRI.

We again complemented our methylene blue dye findings with the added benefits of real-time imaging of a live animal and visualization of the tissue without dissection or manipulation, by performing MR imaging after atraumatically applying 2 ml of gadofoveset trisodium intravaginally in 3 rhesus macaques. We found complete coverage with the contrast agent of the luminal surface of the vagina including the vaginal fornix and the ectocervix, but failed to detect dye in any other site (Fig. 4 and Movie S3). Neither blue dye nor MR contrast methodologies demonstrated any penetration of the agent into the endocervical canal or beyond, regardless of the stage of the female menstrual cycle: follicular, luteal, and actively menstruating. Each animal received no more than 2 ml of dye administered slowly until the lumen was full indicating this to be the maximum capacity of the vagina in these animals. Therefore, the lack of penetration into the cervical canal was not due to a lack of dye or a particular phase of the menstrual cycle but rather due to the normal anatomy and physiology of the cervical os.

### Modeling Lymphatic Drainage from Sites of Intrarectal Inoculation

**Figure 5:**
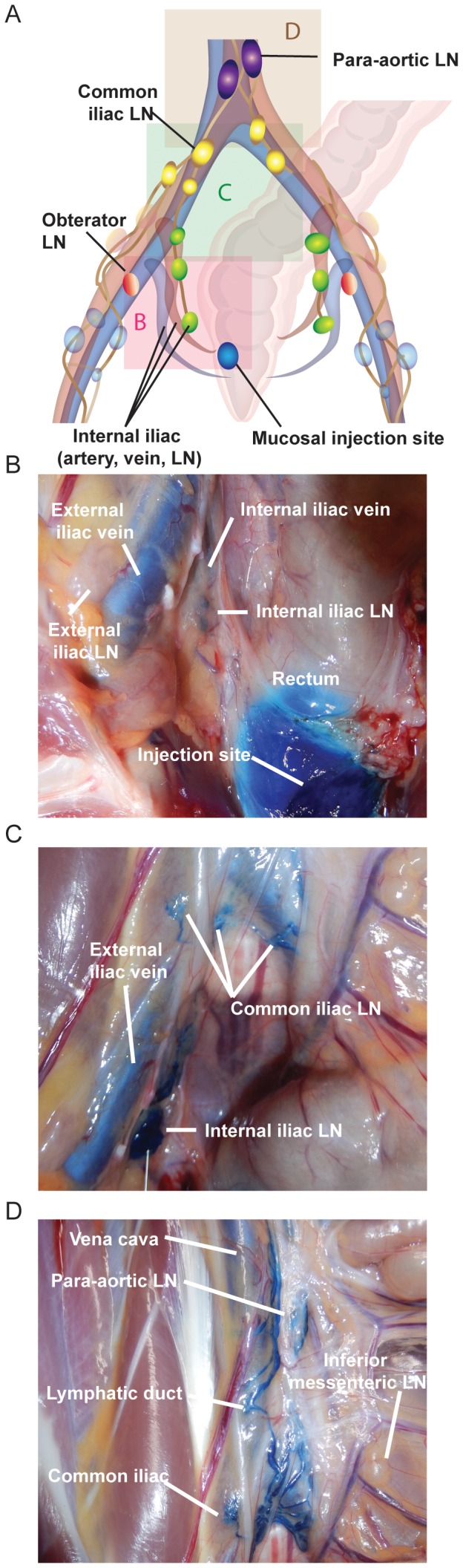
Lymphatic draining patterns following submucosal rectal injections of methylene blue dye. Up to 50 μl of methylene blue was injected submucosally in the rectum prior to euthanasia. During necropsy, stained lymph nodes were identified, photographed and successively reinjected (intranodaly) to identify the next stained lymph node in the draining chain. (A) An illustration of the various pelvic lymph nodes and rectal injection sites for orientation and reference. Each colored box represents the site of each photograph in panels B-D. Panel B shows evidence of dye staining within the internal iliac lymph nodes. Panel C shows evidence of dye staining within the common iliac lymph nodes following internal iliac dye injection, interestingly an anatomic variation in the lymphatic pathway in this animal resulted in dye crossing the midline and enhancing a left common iliac node as well. Panel D shows evidence of dye staining within the para-aortic lymph node following common iliac dye injection. All images are representative of the 5 animals examined by methylene blue dye.

**Figure 6:**
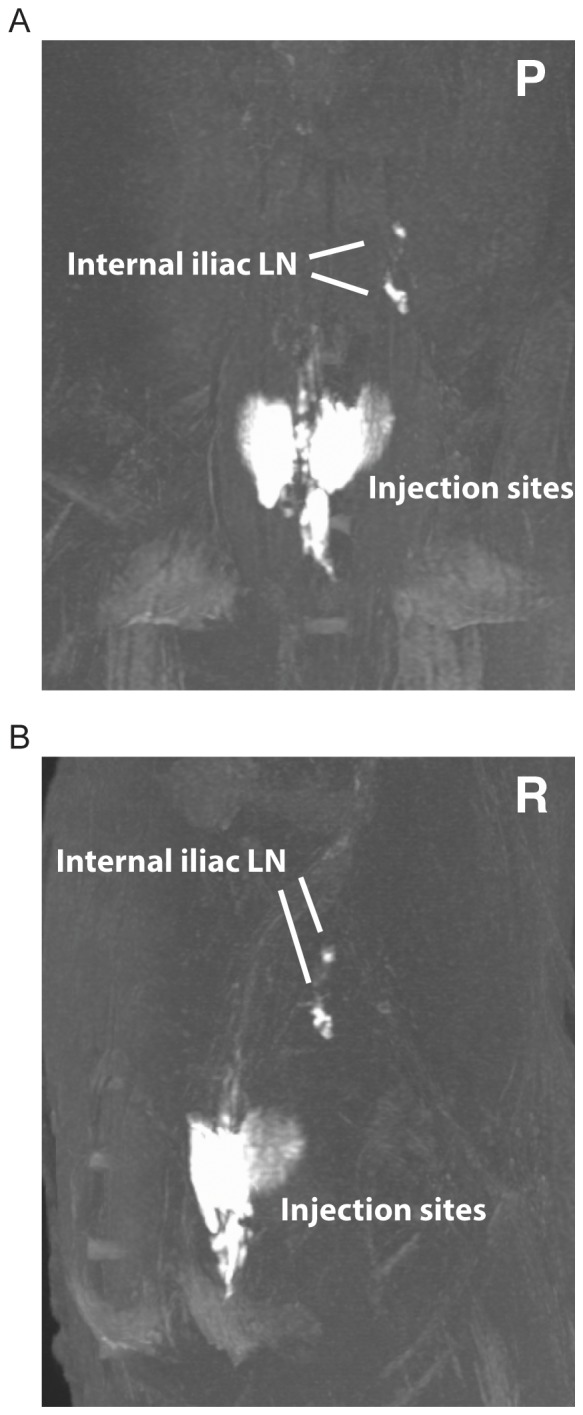
Magnetic resonance imaging to visualize contrast dye following submucosal rectal injections. 50-G5DOTA dendrimer was injected submucosally at two sites within the rectum followed immediately by MR imaging. Within minutes of injection, draining internal iliac lymph nodes were clearly visible. This 3D rendering image is shown in posterior (A) and right view (B) and is representative of the 4 animals examined by MRI.

One essential but unresolved question in HIV/SIV transmission is how the virus or virus infected cell(s) transit from the site(s) of initial local infection at the mucosal portal of entry to distal sites and eventually systemically, to establish a broadly disseminated systemic infection within days of inoculation. To help address this question, we traced the lymphatic drainage patterns from the sites determined by our luminal studies to be potentially important for mucosal transmission. We began by injecting small volumes of methylene blue dye into the submucosa of the rectum, a site consistently exposed to luminal dye following 1 ml or 3 ml intrarectal challenge, just prior to (n = 2) or just following (n = 3) euthanasia. The resulting lymphatic spread was identified during pelvic dissection and illustrated in Figure 5. As shown in Figure 5B, dye trafficked from the mucosa to a draining lymph node within the internal iliac chain. Once the first lymph node was identified, we injected up to 300 μl of dye directly into this node to identify the subsequent lymph nodes within this lymphatic chain draining the rectum. In all instances, the internal iliac node drained into the common iliac chain (Fig. 5C), and from there, into the para-aortic chain (Fig. 5D). Interestingly, we found lymphatic pathways that exhibited significant individual variability in some animals, as demonstrated by the lymphatics crossing the midline and staining both left and right common iliac lymph nodes after injection of the right internal iliac chain with contrast dye (Fig. 5C). We concluded that lymphatic drainage of the rectum was via the internal iliac nodes and that only selected draining nodes are likely to contain infected cells or infectious virus in the early stages of infection, depending on the exact location of viral penetration. Importantly, pararectal lymph nodes were only rarely stained following methylene blue injection, suggesting a limited role for these LN in viral transmission. MRI imaging using Gd-G5-DOTA dendrimer in 4 additional rhesus macaques confirmed these findings, identifying contrast agent within the internal and common iliac nodes following injection in the rectal submucosa (Table 1, Fig. 6 and Movie S4). MR imaging was performed as soon as possible following injection (less than 5min). Importantly, draining lymph nodes were detectable within this short period of time. Utilizing both dye techniques, it is clear that lymphatic drainage from the rectum is first internal iliac, followed by common iliac, and then the para-aortic chain.

**Table 1:** Summary of lymph node staining following submucosal injection of Gd-G5-DOTA dendrimer and MR imaging.

Subject	Injection site	Inguinal LN	External iliac LN	Internal iliac LN	Common iliac LN	Para-aortic LN	Inferior mesenteric LN	Left Colic LN	Para -colonic LN
zj49	Descending Distal Colon	-	-	-	-	+	+	+	+
									
49	Rectum	-	-	+	+	-	-	-	-
									
zg04	Descending Distal Colon	-	-	-	-	+	+	+	+
									
zg04	Rectum	-	-	+	+	-	-	-	-
									
zg83	Rectum	-	-	+	+	-	-	-	-
									
zj04*	Descending Distal Colon	-	-	-	-	+	+	+	+
									
zj04*	Rectum	-	-	+	-	-	-	-	-
									
zg04	Vagina	-	-	+	+	-	-	-	-
									
dc66	Vagina	-	-	+	-	-	-	-	-
									

*20 μl x2 injections.

**Figure 7:**
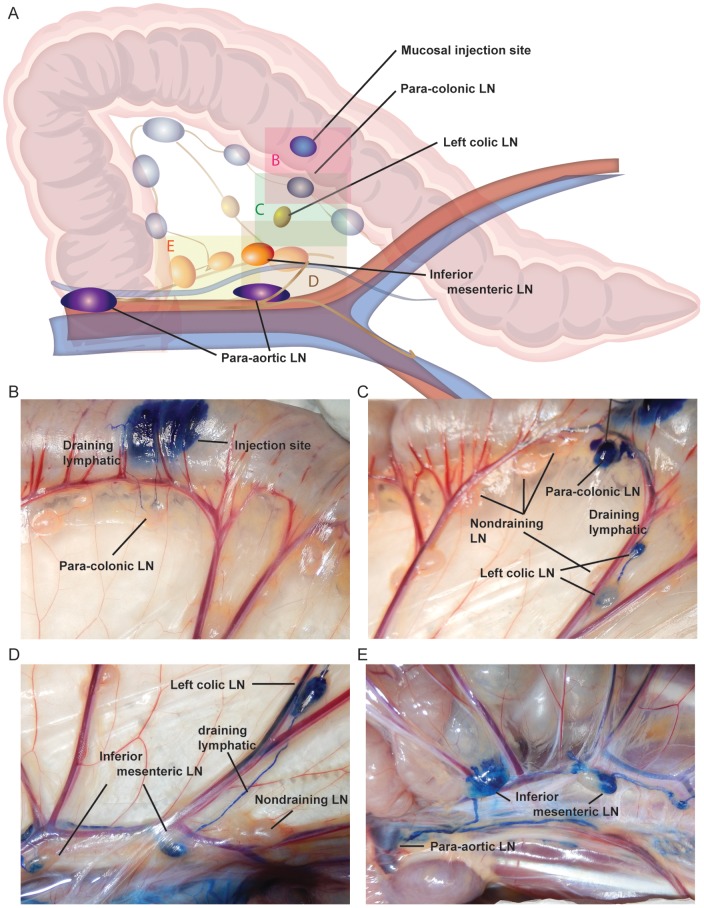
Lymphatic draining patterns following submucosal descending colon injections of methylene blue dye. Up to 50 μl of methylene blue was injected submucosally in the colon following necropsy. Stained lymph nodes were identified, photographed and successively reinjected (intranodaly) to identify the next stained lymph node in the draining chain. (A) An illustration of the various colonic lymph nodes and injection sites for orientation and reference. Each colored box represents the site of each photograph in panels B-E. Panel B shows evidence of dye staining within the para-colonic lymph nodes. Panel C shows evidence of dye staining within the left colic lymph node following para-colonic lymph node injection, note adjacent non-draining lymph nodes. Panel D shows evidence of dye staining within the inferior mesenteric lymph node following left colic lymph node dye injection. Panel E shows evidence of dye staining within the para-aortic lymph node following inferior mesenteric node dye injection. Panels D and E also demonstrate partial staining of an inferior mesenteric lymph node with half completely enhanced by dye and half completely devoid of dye uptake. All images are representative of the 5 animals examined by methylene blue dye.

**Figure 8:**
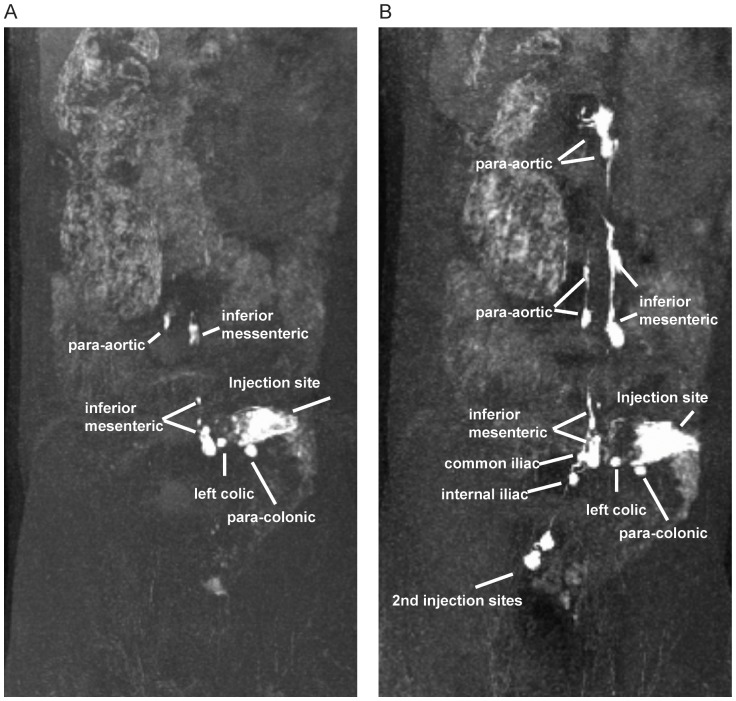
Magnetic resonance imaging to visualize contrast dye following submucosal descending colon and rectal injections. 50 μl of Gd-G5DOTA dendrimer was injected submucosally at two sites within the descending colon (A) followed immediately by MR imaging. Within minutes of injection, draining para-colonic and left colic LN were clearly visible. After several minutes, inferior mesenteric LN and para-aortic LN were detectable. After several imagining sessions, 50 μl of Gd-G5DOTA dendrimer was subsequently injected submucosally at two sites within the rectum of the same animal (B) followed immediately by MR imaging. Within minutes of the second injection, draining internal iliac and common iliac LN were detected. Para-aortic LN demonstrated further enhancement showing the confluence of both routes of drainage. These 3D rendering images are shown only in posterior view and are representative of the 3 animals examined by MRI for colonic lymphatic dissemination.

However, since the luminal exposure to virus as determined by our methylene blue dye and MRI experiments was not confined solely to the rectum, we also determined the lymphatic drainage pattern for the distal descending colon. Methylene blue dye was again injected into the submucosa at the distal descending colon in 5 necropsied rhesus macaques. Lymphatic drainage was observed and photographed following the same approach as for the rectum. From the distal colon, we identified the para-colonic lymph nodes as the first draining node (Fig.7A and B), followed by the left-colic node—when present (Fig. 7C), then to the inferior mesenteric chain (Fig. 7D) and finally again into the para-aortic nodes (Fig.7E). Again, not all adjacent lymph nodes at a given location were dye positive, with a complex web of lymphatic vessels disseminating dye in often unpredictable patterns from the site of dye administration. MRI imaging in sedated live macaques following injection of Gd G5-DOTA dendrimer in the submucosa of the distal descending colon in 3 additional rhesus macaques confirmed these findings. Contrast dye was detected sequentially within the para-colic, left colic, inferior mesenteric, and para-aortic nodes following submucosal injections into the distal descending colon (Table 1, Fig. 8 and Movie S5). Again, MR imaging was performed as soon as possible following injection (less than 5min). Following several imaging sessions of the distal colonic injection, this animal underwent a second submucosal injection of the rectum. MR imaging immediately following the second injection revealed evidence of two distinct lymphatic dissemination patterns based on the location of dye injection (Movie S6). In the same living animal we showed evidence of rectal spread via internal iliac, common iliac and para-aortic chain and from the colon spread from the para-colic, left colic, inferior mesenteric, and para-aortic nodes. Combining the data from both dyes and all animals, it is clear that the distal descending colon drains via the para-colonic and inferior mesenteric lymphatics prior to the para-aortic chain. Although the anatomic boundary between rectum and distal descending colon cannot be precisely determined, it is likely that SIV infection via an intra-rectal challenge would cross this threshold and lymphatic drainage would be either through the iliac chain, or via the colonic lymph nodes.

### Modeling Lymphatic Draining from Sites of Intravaginal Inoculation

**Figure 9:**
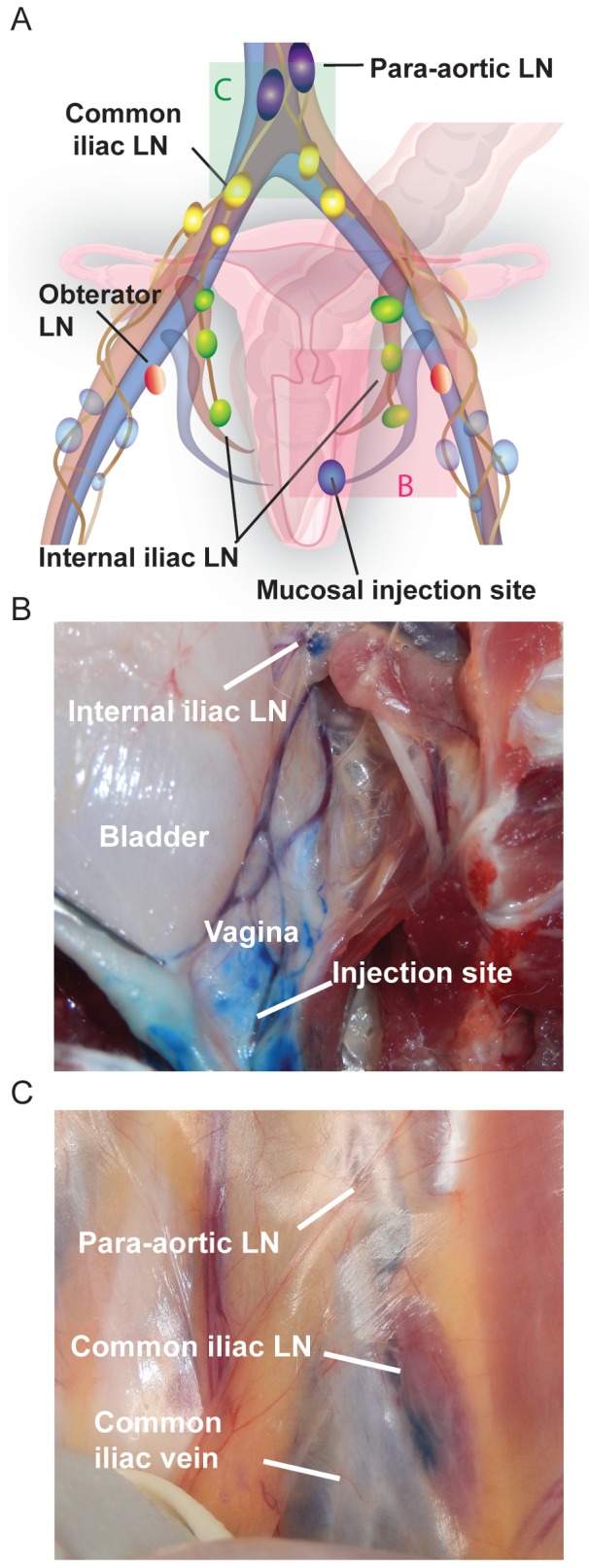
Lymphatic draining patterns following submucosal vaginal injections of methylene blue dye. Up to 50 μl of methylene blue was injected submucosally in the vagina prior to euthanasia. During necropsy, stained lymph nodes were identified, photographed and successively reinjected (intranodaly) to identify the next stained lymph node in the draining chain. (A) An illustration of the various pelvic lymph nodes and vaginal injection sites for orientation and reference. Each colored box represents the site of each photograph in panels B and C. Panel B shows evidence of dye staining within the internal iliac lymph nodes. Panel C shows evidence of dye staining within the common iliac lymph node and para-aortic LN following internal iliac dye injection. All images are representative of the 2 animals examined by methylene blue dye.

**Figure 10:**
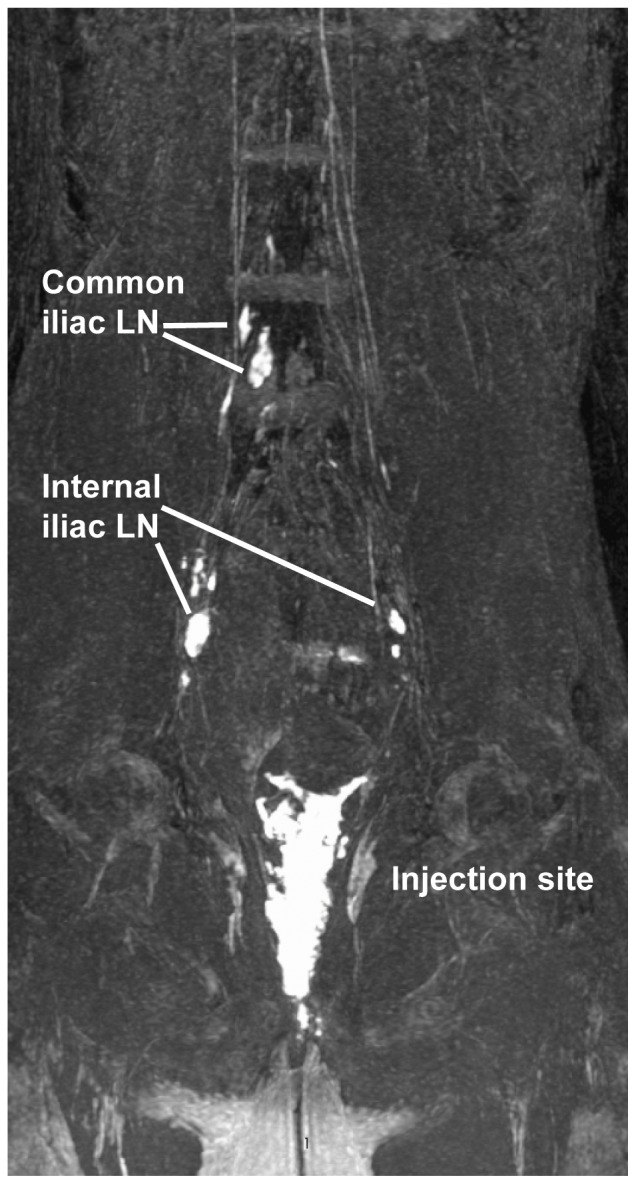
Magnetic resonance imaging to visualize contrast dye following submucosal vaginal injections. 50 μl of Gd-G5DOTA dendrimer was injected submucosally at two sites within the vagina followed immediately by MR imaging. Within minutes of injection, draining internal iliac LN and upstream common iliac LN were clearly visible. This 3D rendering image is shown only in posterior view and is representative of the 2 animals examined by MRI.

By injecting methylene blue dye into the submucosa of the vagina just prior to (n = 1) or just following (n = 1) euthanasia, lymphatic spread was identified again following the same methods employed with the rectum. We discovered that dye trafficked from mucosa to a draining lymph node within the internal iliac chain (Fig. 9A and B). Again, the internal iliac node drained into the common iliac nodal chain (Fig. 9C) as was observed for intrarectal injection. MRI imaging of 2 additional sedated female macaques following injection of Gd G5-DOTA dendrimer in the vaginal submucosa confirmed these findings with detectable contrast dye within the internal and common iliac nodes (Table 1, Fig. 10 and Movie S7). Utilizing both dye techniques, it is clear that lymphatic drainage from the vagina occurs identically to that of the rectum namely via the internal iliac node chain, followed by common iliac, and then the para-aortic lymphatic chain.

### Lymphatic Draining following Intravaginal SIV Infection

**Figure 11:**
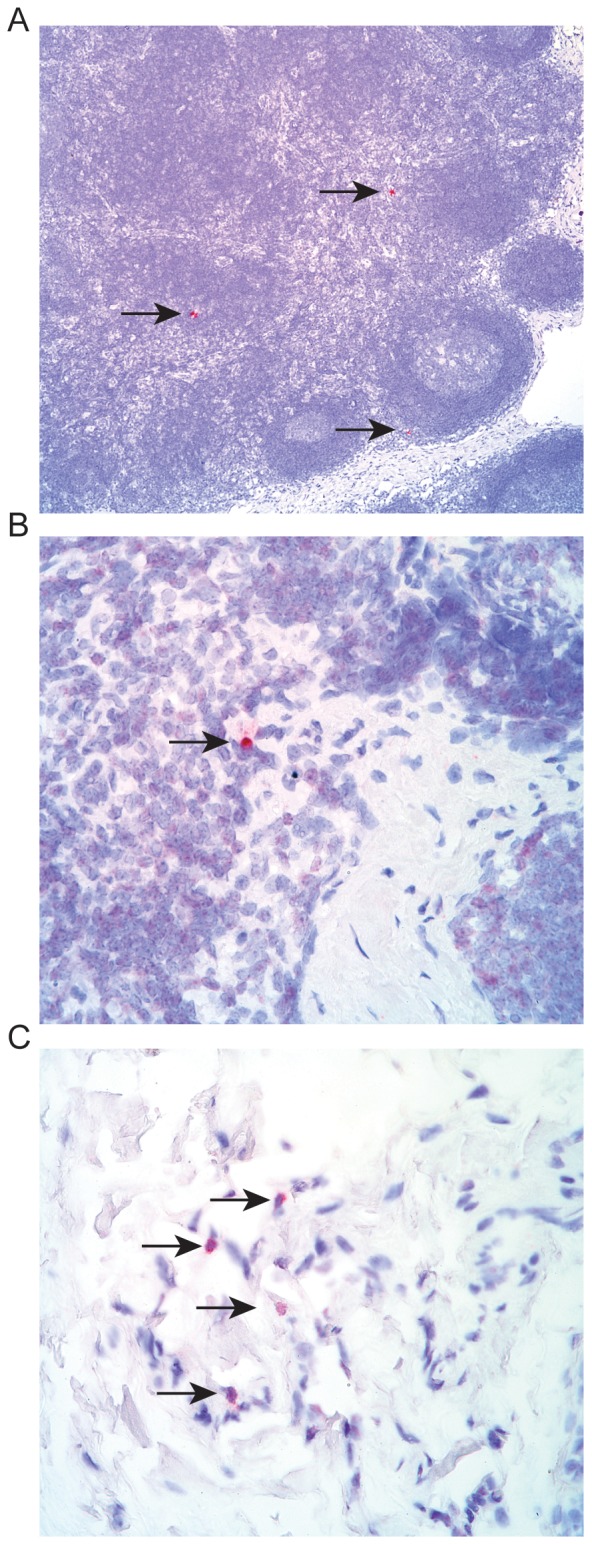
In situ hybridization positive cells within the draining internal iliac LN 3 days following vaginal challenge with SIVmac. A. Low magnification of an internal iliac lymph node section showing three productively infected cells with lymphocytic morphology (arrows). B. High magnification of a productively infected cell with lymphocytic morphology (arrow) in the paracortical T cell zone adjacent to a trabecular sinus. C. High magnification of four cells harboring SIV vRNA with a dendritic cell morphology (arrows) within the capsular sinus, which is consistent with cells (putatively DCs) bearing SIV immigrating into the internal iliac lymph node.

To assess the relevance of the results of these studies employing simulated inocula to actual viral transmission, we evaluated the localization of SIV infection following intravaginal exposure to an infectious SIV inoculum and compared our findings to the spread of non-infectious simulated dye inocula. Three days following intravaginal challenge with SIVmac239, tissues including para-aortic, inguinal, axillary, bronchial and mandibular lymph nodes, liver, spleen, bone marrow, upper and lower gastrointestinal tract, and the entire female reproductive tract were obtained at necropsy and subjected to in situ hybridization to detect the presence of viral RNA. The only tissue outside of the vagina/cervix that was positive by in situ hybridization was an internal iliac lymph node (Fig. 11). Here we detected both productively infected cells as well as viral RNA in the afferent capsule of the iliac lymph node suggesting early viral spread via the lymphatic system and a pathway consistent with our model. Importantly, neighboring lymph nodes were ISH negative as were sham challenged animals confirming the specificity of this assay and reaffirming the variable but selective nature of the lymphatics that can serve as potential conduits for viral dissemination.

## Discussion

Recently, genetic analyses of acute HIV infections have shown that over 80% of typical sexual transmissions originate from a single viral variant from among the diverse quasispecies typically represented in the donor. This has resulted in a shift in paradigm for viral challenges in NHP studies, especially vaccine studies, to designs that attempt to recapitulate this key feature of HIV infection. Given the diversity present in a typical viral inoculum and the fact that the selected variant is highly variable, this single variant infection likely results from a single founder virus. Other variants within the inoculum may be stopped at various stages before or after crossing the mucosal barrier, but before establishment of systemic viremia. Successfully elucidating these early events requires an understanding of where virus entry occurs and how the infection disseminates from these sites of entry.

To begin understanding this essential phase in viral transmission, we used simulated viral inoculations administered intravaginally and intrarectally, using methylene blue dye or gadofoveset trisodium with MR imaging to: (i) better define the current mucosal challenge approaches employed in nonhuman primates to model the two most common modes of HIV transmission (vaginal- and receptive, anal-intercourse); and, (ii) to refine these models to reduce potential confounding variables which may improve the reproducibility and robustness of these models and our ability to study transmission and early events. Initially, we used these simulated challenges to ascertain which mucosal surfaces come into contact with typical inocula in order to identify potential anatomical sites of virus entry across mucosal barriers.

Administration of 1 or 3 ml of MRI contrast dye or vital dye intrarectally often spread through the rectum to the distal descending colon, identifying these two segments of lower GI tract as potential sites of viral entry. Given the typical length of a human rectum (∼12 cm) [19] it is likely that these same anatomical locations are relevant to human transmission. Importantly, the 1 ml challenge was inconsistent in reaching the distal descending colon, and therefore might serve to only model rectal exposure to virus in roughly half of the animals. This has the potential to increase variation between animals infected at different sites and limiting the usefulness of single lymph node analysis following infection. For most challenge studies, consistency of exposure animal-to-animal is paramount, as either the rate of acquisition is being evaluated or there is a need for all animals to become infected at the same time but with limited numbers of variants transmitted. To this end a 3 ml IR challenge inoculum would appear likely to increase the consistency of contact between the mucosa and the inoculum. Further, a typical macaque ejaculate is 2.7 +/– 1 ml [20], which is consistent with a 3 ml inoculum volume. Finally, the surface area covered by a 3 ml volume in this small animal model is likely more consistent with inoculum coverage in humans following receptive anal-intercourse, though neither our model nor typical viral challenge models take into account potential effects of differences in the viscosity of the material inoculated compared to semen.

Additional factors evaluated that could affect the exposure of the mucosa and thereby the target cells accessed during IR challenge included the amount and consistency of feces, and the position of the animal during inoculation. Feces greatly impacted the amount of mucosal exposure to simulated rectal inoculation, with larger amounts of feces leading to decreased mucosal dye staining, although this effect was largely mitigated by using a larger simulated inoculum volume. The consistency of the fecal material also had an effect, with firm feces leading to complete exclusion of the dye from certain areas of the mucosa, whereas softer feces led to a mixing of the feces with the dye and a lighter staining of the mucosa as a result of this dilution effect. The positional angle of the animal had no apparent effect on the consistency of mucosal coating, but a lower angle appeared to result in a slight though not statistically significant reduction in the distance the dye traveled from the anal verge (data not shown). A number of other factors not evaluated in this study might also have the potential to impact viral challenges including (i) the choice of anesthetic and the resultant effects on peristalsis, (ii) the use of enemas resulting in potential residual volumes of liquid which could dilute the inoculum, (iii) physical methods to remove fecal matter that may result in mucosal damage; and (iv) the impact of various enteric pathogens with the potential to affect mucosal integrity, fluid absorption, and inflammatory state, which could impact both the spread of the inoculum and the presence and activation state of target cells. Careful consideration of these parameters could further decrease variability between studies and provide more consistent viral challenges.

Simulated dye inoculations were also used to evaluate intravaginal challenges. Administration of 2 milliliters of MRI contrast/vital dye intravaginally identified the vagina and ectocervix as possible sites of viral entry but failed to demonstrate exposure to the endocervix, uterus or any other tissues in the female reproductive system. In our simulated vaginal inoculations neither the position of the animal during dye administration, nor the phase of the menstrual cycle had an effect on the penetration of the dye into the cervical canal and subsequent exposure of more proximal portions of the female genital tract. However the contrast dye, and the typical viral inoculum it is intended to model, moves by passive diffusion, which may not represent what would occur during sexual intercourse. There is also concern that intercourse during the menstrual phase could lead to greater risk of transmission both female to male and male to female. Many studies in nonhuman primates either avoid the menstrual phase or do not account for the menstrual cycle when performing viral challenges. Other potential factors such as the presence of estral mucus and effects of previous parturition could also have an effect on the locations and amount of mucosa exposed to the challenge inoculum, but these were not evaluated in this study.

After identifying the anatomical sites of inoculum interaction and presumptive initial viral infection, we then targeted these sites for submusosal injections with Gd G5 DOTA dendrimer followed by MRI or methylene blue followed by necropsy to define patterns of lymphatic draining and by inference, the likely pathway of virus dissemination. The effectiveness and accuracy of determining the draining lymphatic pathways using dyes has been studied extensively and is currently used by surgeons to identify sentinel nodes in cancer patients [21]. In fact Bostick et al. reported that “the blue dye technique remains the criterion standard for Sentinel Lymph Node Detection (SLND) in melanoma” [22]. To our knowledge, this study represents the first attempt to map the lymphatic drainage of the vagina, rectum, and distal descending colon in macaques using these combined approaches. Gd G5 DOTA dendrimer was selected for this purpose based on its size (∼8 nm) and the high density of Gd on its surface. This results in a nanoparticle that has high relaxivity, making it easier to detect by MRI. At the same time, the physical properties of the nanoparticles are ideal for uptake by the lymphatics and once taken up, the contrast material appears to be confined within the lymphatic system. Gadofosveset is transiently bound to albumin giving it a high relaxivity, however, in its native, unbound form it is a low molecular weight agent that is not contained within the lymphatics and thus is inferior to Gd-G5-DOTA dendrimer for tracking lymphatic spread.

**Figure 12:**
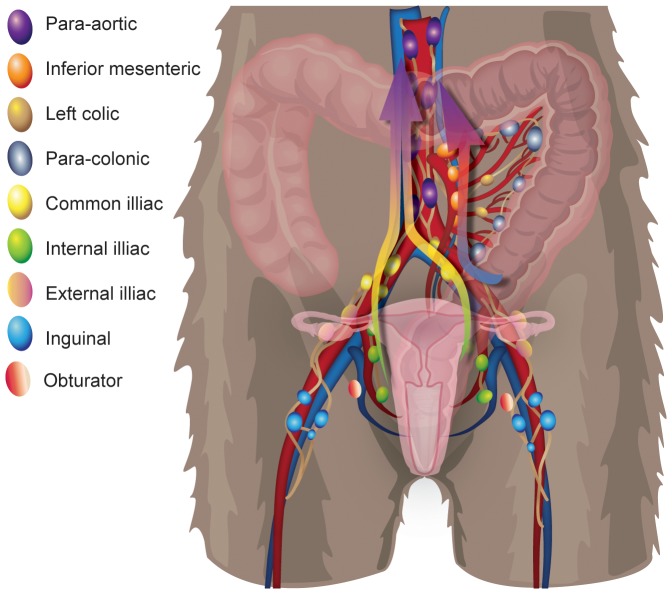
Cartoon illustration representing the two distinct pathways for lymphatic dissemination from the sites of mucosal exposure. The anatomic position and commonly used nomenclature for all relevant lymphatic nodes are shown. The two distinct lymphatic dissemination pathways are highlighted by arrows with rectal/vaginal dissemination pathway occurs via the internal iliac, then the common iliac followed by the para-aortic chain. The colonic dissemination pathway includes the para-clonic LN, the left colic LN, inferior mesenteric LN, and the para-aortic system.

Injecting Gd G5 DOTA dendrimer submucosally in the vagina (∼1.5–3 cm from the vaginal introitus), the rectum (∼1.5–3 cm from the anal verge), and the descending colon (∼10–13 cm from the anal verge) resulted in contrast dye detection within draining lymph nodes. There were two clear and distinct pathways for passive lymphatic diffusion identified with MRI and visually with methylene blue dye. The first pathway involved entry within the descending colon with para-colonic nodes as the initial site of dye uptake, followed by the left colic nodes (when present), inferior mesenteric, and then the para-aortic nodes (Fig. 12). The second pathway involved entry within the rectum or vagina with internal iliac lymph nodes as the initial site of dye uptake, followed by the common iliac nodes and then the para-aortic nodes (Fig. 12). In humans and most likely in macaques the lymphatic drainage would proceed from the para-aortic chain to the cisterna chyli, thoracic duct and then into the systemic circulation via the left subclavian vein. The lymphatic anatomy of the macaque in this area is very similar to human anatomy and drainage pathways identified are very similar to those identified in humans using MR imaging [23]. The only major difference was that a number of human anatomical references, including Gray’s anatomy [19], show lymphatic drainage of the vagina and distal rectum to the inguinal and external iliac nodes, however we found no evidence of this in our macaques. It is important to note that regardless of the site of injection not all nodes at each anatomic site demonstrated contrast uptake, with adjacent nodes showing either complete staining or complete lack of staining based on the complex and variable web of afferent lymphatic ducts which can be identified only when using dyes. In some cases, half of a node was completely stained and the other half was entirely free of staining, demonstrating how selective these drainage patterns are. These half-stained nodes may represent two adjacent nodes that fused, leaving the gross appearance of a single node or separate compartments of the same node that drain different afferent pathways. Furthermore, in the colonic pathway a variable number of distal para-aortic nodes were bypassed based on where the inferior mesenteric lymphatics first connected to the para-aortic pathway. This variability of contrast uptake could translate into an equivalent variability in the presence or absence of virally infected cells in adjacent nodes during the early stages of viral dissemination following intrarectal or intravaginal viral challenge. In fact, this is precisely what we observed in an acutely infected animal following vaginal challenge. Intravaginal infection with SIVmac239, followed by euthanasia at 3 days with extensive tissue examination by ISH identified the presence of infection following the same pathway as the injected contrast agents and with the same localization to individual nodes at a given site as were identified using a simulated challenge. Others have attempted to look at early dissemination of virus following vaginal inoculation with limited success in identifying infected local nodes prior to viremia; perhaps as a result of the localized and variable nature of lymphatic drainage identified here [24]. Furthermore, the sentinel or draining lymph nodes were not assessed in this prior study, which was instead focused on the mucosal surface and distal lymph nodes. Others have also studied mucosal transmission using GFP labeled virus and determining mucosal penetration of virus with no capability to identify patterns of dissemination from the sites of transmission [25], [26]. Although both approaches are informative and flank our study both anatomically and temporally, here we sought to connect the mucosa to distal sites by determining the likely routes of systemic dissemination starting with the earliest lymphatic tissues the virus would encounter.

Based on the likelihood of localized infection of the rectum/colon and the local draining nodes during the early pre-viremia phase, when critical events are occurring that will shape the course of the infection, extensive sampling of the distal 15–18 cm of the colon/rectum and all identified nodes (internal iliacs, common iliacs, para-colonics, left colics, inferior mesenteric and para-aortic nodes may be necessary to ensure that the samples taken are adequate to accurately analyze these early events. Given that viral entry likely occurs at one or a very limited number of foci in the vagina/ectocervix or rectum/descending colon using a limited dose challenge, partial sampling via pinch biopsies or small sectional sampling is unlikely to fully capture and permit analysis of early events that lead to infection and previremic dissemination. Additionally, it may be necessary to analyze all lymph nodes individually in these critical regions or use a labeling methodology that allows for visualization of virally infected cells at the time of sample collection to ensure proper sampling. More distal sites, such as the para-aortic nodes, that drain larger regions of the inoculated organ may be more appropriate specimens if sampling later after challenge. Alternatively, employing a means of localizing the entry point of infection to a reproducible and identifiable region may facilitate collection of appropriate samples. These findings may also be useful in vaccine strategies that administer antigen mucosally to generate a more focused immune response that may improve vaccine efficacy. Understanding which draining lymph nodes and likely routes of viral transmission would be important in designing mucosal vaccines. Furthermore, testing sites and tissues known to participate in SIV/HIV infection prior to challenge may be important to determine the likelihood of successful immune intervention regardless of how vaccine was administered.

In addition to their relevance for studies of retroviral infection, these results have profound implications for the utilization of MRI technology to map sentinel nodes, as is often performed for staging cancer in patients. Currently, solid tumors are injected with radiolabeled sulfur colloid and radioactivity is traced to seminal nodes using a handheld gamma probe. The MRI based technique implemented in this study could potentially be employed similarly in cancer patients, with the advantages of avoiding radiation exposure for the patient and better anatomical precision with superior soft tissue resolution for presurgical preparation if the contrast agent could be injected into tumors under imaging guidance.

In conclusion, this study demonstrates a methodology for assessing the coverage of an inoculum within the distal colon, rectum and vagina, and it employs that methodology to evaluate and suggest refinements to nonhuman primate models of vaginal- and receptive, anal-intercourse transmission of HIV. Moreover, it demonstrates a methodology for identifying the specific lymphatic drainage system based on the precise sites of infection likely to occur in a given model. The macaque anatomy pertinent to this study was remarkably similar to and should serve as an appropriate model for human anatomy relevant to rectal and vaginal transmission and lymphatic dissemination of HIV. Injection of contrast dye identified pathways that were consistent with viral infection following vaginal challenge with SIVmac239. This model predicts that initial spread of the virus from the site of entry occurs lymphatically with profound implications for sampling and studying early events of viral transmission. Finally, as a practical matter, the study demonstrated that a 3 ml intrarectal challenge volume results in more consistent coating of the mucosa which would likely result in more consistent exposure of virus to target cells in studies where consistent infection is critical.

## Supporting Information

Figure S1 Methylene blue dye staining of female genital tract following a 2 ml inoculum challenge. Twenty minutes following dye exposure, actively menstruating animal were necropsied and the entire female genital tract was extracted en block and dissected. The tissue was photographed to assess both completeness of dye coverage and length of distance from the vaginal introitus. Overall there was complete dye staining in the vaginal vault, but stain was not detected in the cervix, uterus, or ovaries.
(TIF)

Movie S1 Maximum intensity projections representing a 3D reconstruction of magnetic resonance imaging to visualize contrast dye introduced intrarectally in a rhesus macaque exposed to a 1 ml inoculum. Gadofoveset trisodium contrast dye was administered intrarectally and 20 minutes later animals were returned to a recumbent position and MRI was performed. The 1 ml exposure shown here shows robust inoculum penetration including within the distal descending colon. Although background contrast was detected in the small intestines due to animal diet, contrast dye in the rectum and descending colon is clearly visible. This movie was selected to represent the 3 animals tested by MRI for luminal coverage following a 1 ml exposure.
(MP4)

Movie S2 Maximum intensity projections representing a 3D reconstruction of magnetic resonance imaging to visualize contrast dye introduced intrarectally in a rhesus macaque exposed to a 3 ml inoculum. Gadofoveset trisodium contrast dye was administered intrarectally and 20 minutes later animals were returned to a recumbent position and MRI was performed. The 3 ml exposure shown here shows robust inoculum penetration including within the distal descending colon. Bladder compression of the distal descending colon results in a thin layer of dye between these two sites, which, though difficult to see, likely coats the compressed mucosa. Although background contrast was detected in the small intestines due to animal diet, contrast dye in the rectum and descending colon is clearly visible. This movie was selected to represent the 2 animals tested by MRI for luminal coverage following a 3 ml exposure.
(MP4)

Movie S3 Maximum intensity projections representing a 3D reconstruction of magnetic resonance imaging to visualize contrast dye introduced intravaginally in a rhesus macaque. 2 ml of Gadofoveset trisodium contrast dye was applied intravaginally and 20 minutes later animals were returned to a recumbent position and MRI was performed. This movie demonstrates the complete saturation of the vaginal vault and no contrast penetration into the cervix/uterus despite the high degree of anatomic resolution that can be achieved by MRI. This movie was selected to represent the highly consistent vaginal exposure in all 4 animals examined by MRI.
(MP4)

Movie S4 Maximum intensity projections representing a 3D reconstruction of magnetic resonance imaging to visualize contrast dye introduced submucosally within the rectum. 50 μl of Gd-G5DOTA dendrimer was injected submucosally in the rectum. Within minutes of injection, the draining internal iliac LN and common iliac LN were clearly visible. This movie was selected to represent the 4 animals examined by MRI for rectal lymphatic dissemination.
(MP4)

Movie S5 Maximum intensity projections representing a 3D reconstruction of magnetic resonance imaging to visualize contrast dye introduced submucosally within the colon. 50 μl of Gd-G5DOTA dendrimer was injected submucosally at two sites within the distal descending colon followed immediately by MR imaging. Within minutes of injection, the draining para-colonic and left colic LN were clearly visible. This movie was selected to represent the 3 animals examined by MRI for colonic lymphatic dissemination.
(MP4)

Movie S6 Maximum intensity projections representing a 3D reconstruction of magnetic resonance imaging to visualize contrast dye introduced submucosally within the colon and subsequently within the rectum also. 50 μl of Gd-G5DOTA dendrimer was injected submucosally at two sites within the distal descending colon followed immediately by MR imaging. Within minutes of injection, the draining para-colonic and left colic LN were clearly visible. After several imagining sessions, 50 μl of Gd-G5DOTA dendrimer was subsequently injected submucosally at two sites within the rectum of the same animal followed immediately by MR imaging. Within minutes of the second injection, draining internal iliac and common iliac LN were detected. Para-aortic LN demonstrated further enhancement showing the confluence of both routes of drainage. These 3D rendering images are shown only in posterior view and are representative of the 5 animals examined by MRI. This movie was selected to represent the 7 animals examined by MRI for rectal and colonic lymphatic dissemination.
(MP4)

Movie S7 Maximum intensity projections representing a 3D reconstruction of magnetic resonance imaging to visualize contrast dye introduced submucosally within the vagina. 50 μl of Gd-G5DOTA dendrimer was injected submucosally at two sites within the vagina followed immediately by MR imaging. Within minutes of injection, draining internal iliac LN and upstream common iliac LN were clearly visible. This movie was selected to represent the 2 animals examined by MRI for vaginal lymphatic dissemination.
(MP4)
