# Correction: Tracking the Luminal Exposure and Lymphatic Drainage Pathways of Intravaginal and Intrarectal Inocula Used in Nonhuman Primate Models of HIV Transmission

**DOI:** 10.1371/journal.pone.0288566

**Published:** 2023-07-07

**Authors:** Jeremy Smedley, Baris Turkbey, Marcelino L. Bernardo, Gregory Q. Del Prete, Jacob D. Estes, Gary L. Griffiths, Hisataka Kobayashi, Peter L. Choyke, Jeffrey D. Lifson, Brandon F. Keele

The 13th sentence of the first paragraph of the Materials and Methods subsection Animals, regarding the antibiotic/antiparasitic treatment regimen, is incorrect. The correct text is: Prior to the study and as part of the facility animal management, 22 animals received an oral antibiotic/antiparasitic regimen between 1 and 10 months prior to study which included metronidazole (50 mg + 21mg/kg or 50 mg/kg once or twice daily for 5–10 days) and fenbendazole (50 mg/kg once daily for 3–5 days), with some of these animals (n = 15) also receiving enrofloxacin (10 mg/kg once daily for 7 days). One animal received paromomycin (50 mg/kg twice daily for 12 days) and two cycles of fenbendazole (50 mg/kg once daily for 5 days) with the final animal receiving only enrofloxacin (5 mg/kg intramuscularly once daily for 7 days).

Individual-level treatment information for the 24 study animals is provided here in S1 File.

The authors are unable to independently confirm that animals were subjected to weekly fecal culture and parasite exams for 3 weeks.

There is an error in the labelling of Fig 1A: animal ID 4450 is incorrect; the correct ID is 4550. A corrected Fig 1 is provided below.

**Fig 1:**
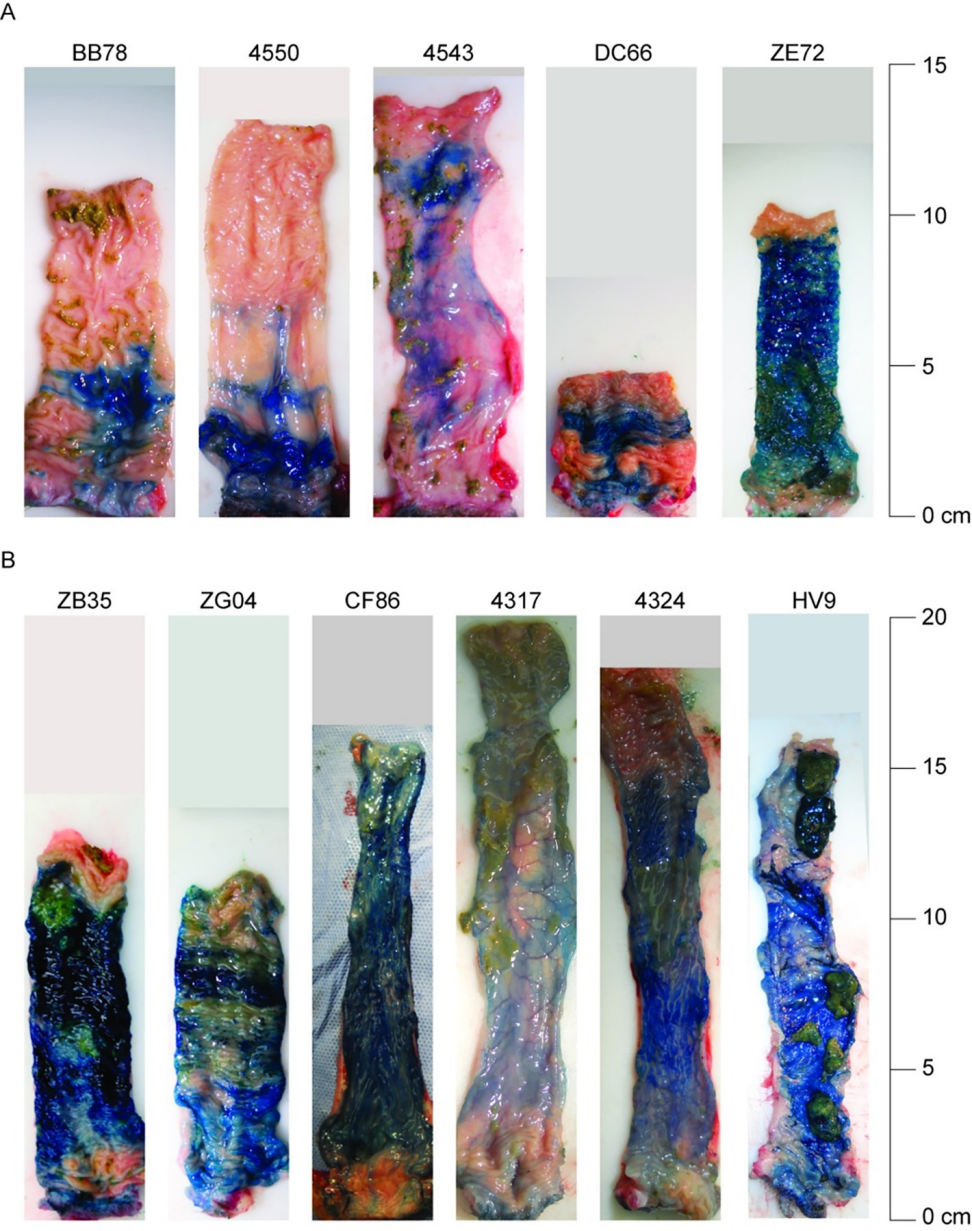
**Methylene blue dye staining of rectum and descending colon following a 1 ml (A) or 3 ml (B) simulated challenge.** Twenty minutes following dye exposure, animals were necropsied and rectum/distal colon was removed en block. Once fecal matter was gently cleared from tissue, stained mucosal tissue was photographed to assess both completeness coverage and the maximal distance of dye coverage from the anal verge.

There are errors in the first column of Table 1. The subject IDs in the top two rows, zj49 and 49, are incorrect; the correct ID for both is zj47.

This study involved the use of 24 rhesus macaques and visible dye or non-invasive MRI to mimic the luminal exposure and lymphatic draining pathways following vaginal or rectal viral challenge. The authors do not believe that the differences in the antibiotic/antiparasitic treatment used prior to the study affected the results and conclusions.

## Supporting information

S1 File Treatment information. This file includes treatment information for each study animal.
(PDF)
