# Supplementary figures and images for: Tracking the Luminal Exposure and Lymphatic Drainage Pathways of Intravaginal and Intrarectal Inocula Used in Nonhuman Primate Models of HIV Transmission
Source: PLoS One. 2014 Mar 25;9(3):e92830. doi: 10.1371/journal.pone.0092830 (PMC3965472; doi:10.1371/journal.pone.0092830)

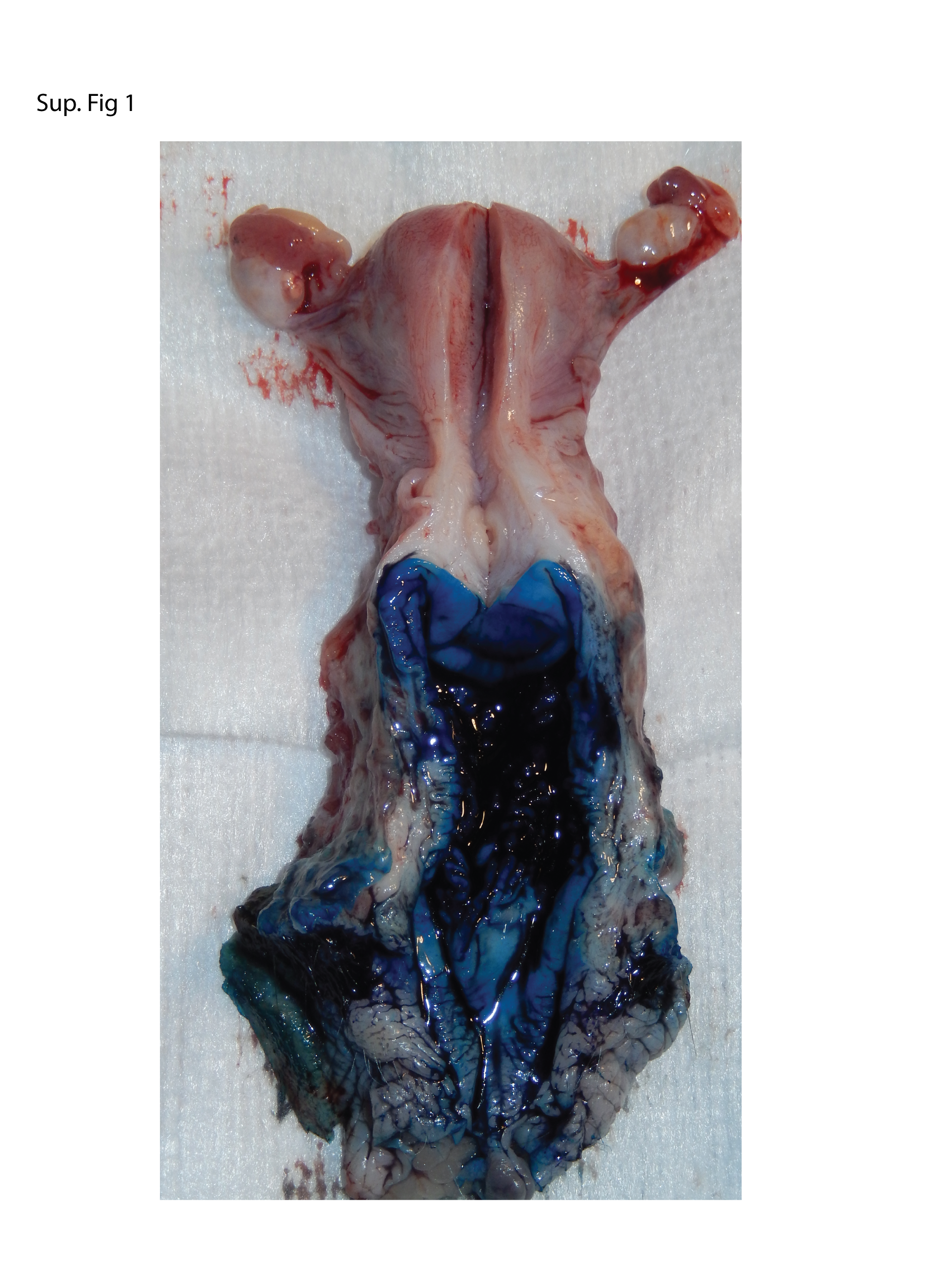

Supplement: Figure S1 — Methylene blue dye staining of female genital tract following a 2 ml inoculum challenge. Twenty minutes following dye exposure, actively menstruating animal were necropsied and the entire female genital tract was extracted en block and dissected. The tissue was photographed to assess both completeness of dye coverage and length of distance from the vaginal introitus. Overall there was complete dye staining in the vaginal vault, but stain was not detected in the cervix, uterus, or ovaries. (TIF) [file pone.0092830.s001.tif]
