# Supplementary material for: Correction: Tracking the Luminal Exposure and Lymphatic Drainage Pathways of Intravaginal and Intrarectal Inocula Used in Nonhuman Primate Models of HIV Transmission
Source: PLoS One. 2023 Jul 7;18(7):e0288566. doi: 10.1371/journal.pone.0288566 (PMC10328372; doi:10.1371/journal.pone.0288566)
Supplement: S1 File — This file includes treatment information for each study animal. (PDF) [file pone.0288566.s001.pdf]

Animal I.D.	Gender	Rectum Intraluminal *MB 1ml (n=5)	Rectum Intraluminal *MB 3ml (n=6)	Vagina Intraluminal *MB (n=6)	Rectum Submucosal *MB (n=5)	Colon Submucosal *MB (n=5)	Vagina Submucosal *MB (n=2)	Rectum Intraluminal MRI 1ml (n=3)	Rectum Intraluminal MRI 3ml (n=2)	Vagina Intraluminal MRI (n=3)	Rectum Submucosal MRI (n=4)	Colon Submucosal MRI (n=3)	Vagina Submucosal MRI (n=2)	Vaginal Infection (n=1)	Baytril (Enrofloxacin)	Metronidazole	Panacur (Fenbendazole)	Time between treatment and study
4550^	M	√														50mg+21mg/kg PO BID x 10 days	50mg/kg PO SID x 5 days	3 months
4543	M	√														50mg+21mg/kg PO BID x 10 days	50mg/kg PO SID x 5 days	3 months
4317	M		√													50mg+21mg/kg PO BID x 10 days	50mg/kg PO SID x 5 days	3 months
4324	M		√													50mg+21mg/kg PO BID x 10 days	50mg/kg PO SID x 5 days	3 months
HV9	F		√														50mg/kg PO SID x 5 days #	8 months
CF86	M		√												10mg/kg PO SID x 7 days	50mg/kg PO SID x 5 days	50mg/kg PO SID x 3 days	4 months
986	F			√											10mg/kg PO SID x 10 days	50mg/kg PO BID x 10 days	50mg/kg PO SID x 5 days	4 months
ZB35	F		√	√												50mg/kg PO BID x 10 days	50mg/kg PO SID x 5 days	10 months
BB78	F	√		√												50mg/kg PO BID x 10 days	50mg/kg PO SID x 5 days	1 month
ZE72	F	√		√											10mg/kg PO SID x 7 days	50mg/kg PO SID x 5 days	50mg/kg PO SID x 3 days	7 months
DC66	F	√		√						√			√			50mg/kg PO BID x 10 days	50mg/kg PO SID x 5 days	6 months
ZG04	F		√	√				√	√	√	√	√	√		10mg/kg PO SID x 7 days	50mg/kg PO SID x 5 days	50mg/kg PO SID x 3 days	3 months
ZG83	M							√			√				10mg/kg PO SID x 7 days	50mg/kg PO SID x 5 days	50mg/kg PO SID x 3 days	3 months
ZJ04	M								√		√	√			10mg/kg PO SID x 10 days	50mg/kg PO SID x 10 days	50mg/kg PO SID x 3 days	2 months
ZJ47^^	M										√	√			10mg/kg PO SID x 10 days	50mg/kg PO SID x 10 days	50mg/kg PO SID x 3 days	2 months
ZJ63	F						√	√		√					10mg/kg PO SID x 10 days	50mg/kg PO SID x 10 days	50mg/kg PO SID x 3 days	2 months
ZD28	F						√								10mg/kg PO SID x 7 days	50mg/kg PO SID x 5 days	50mg/kg PO SID x 3 days	3 months
ZH08	M				√	√									10mg/kg PO SID x 7 days	50mg/kg PO SID x 5 days	50mg/kg PO SID x 3 days	9 months
ZH39	M					√									10mg/kg PO SID x 7 days	50mg/kg PO SID x 5 days	50mg/kg PO SID x 3 days	9 months
Zi23	M				√	√									10mg/kg PO SID x 7 days	50mg/kg PO SID x 5 days	50mg/kg PO SID x 3 days	9 months
ZG20	M				√	√									10mg/kg PO SID x 7 days	50mg/kg PO SID x 5 days	50mg/kg PO SID x 3 days	9 months
Zi47	M				√										10mg/kg PO SID x 7 days	50mg/kg PO SID x 5 days	50mg/kg PO SID x 3 days	9 months
Zi52	M				√	√									10mg/kg PO SID x 7 days	50mg/kg PO SID x 5 days	50mg/kg PO SID x 3 days	9 months
A7E018	F													√	5mg/kg IM SID x 7 days			8 months

* Methylene blue dye administration

^ "4550" is correct animal ID. Incorrectly identified as "4450" in Figure 1.

^^ "ZJ47" is correct animal ID. Incorrectly identified as "ZJ49" and "49" in Table 1.

Two series of Fenbendazole + Paromomycin (50mg/kg PO BID x 12 days)
